# Balancing Professional Commitments and Personal Life for Women in Interventional Radiology

**DOI:** 10.1007/s00270-025-04103-w

**Published:** 2025-06-24

**Authors:** Georgia Tsoumakidou

**Affiliations:** Department of Diagnostic and Interventional Radiology, Lausanne University Hospital and University of Lausanne, Rue du Bugnon 46, 1011 Lausanne, Switzerland

**Keywords:** Work life balance, Interventional radiology, Gender equity, Burn out prevention, Mentorship

## Abstract

Interventional Radiology (IR) is a captivating yet demanding specialty, characterized by its technical complexity, time-sensitive procedures, and intensive work environment. Women in IR face additional challenges, including underrepresentation, implicit bias, limited mentorship and the struggle to balance professional and personal responsibilities. The long, unpredictable working hours, the on call duties and the physical demands of the specialty further complicate work-life integration. This article explores key strategies to create a more inclusive and supportive environment, promoting sustainable careers and advancing gender equality in the field.

## Introduction

The concept of work–life balance has its roots in nineteenth-century labor reforms and evolved through milestones like the 44-h workweek and the women’s liberation movement [1]. By the late twentieth century, attention shifted toward balancing professional and personal spheres, popularized by pioneers like Lillian Gilbreth [2], who emphasized the importance of integrating well-being with work productivity.

In health care, and particularly in interventional radiology (IR), work–life balance is a pressing issue—especially relevant for women. While IR offers unique opportunities and career paths, it also demands long hours, on-call responsibilities, and a need for emotional resilience. This article explores the challenges women face in IR and highlights strategies for fostering a more supportive and equitable environment. By addressing these issues, we aim to promote sustainable careers and advance gender equality in the specialty.

## The Demands and Challenges of Interventional Radiology

IR is a captivating yet demanding specialty, characterized by its technical complexity, time-sensitive procedures, and intensive work environment. It requires IR practitioners to possess advanced technical skills, precise coordination, and the ability to make rapid decisions under pressure—often in urgent scenarios such as trauma, stroke, or sepsis management. The work is further intensified by long, unpredictable hours, frequent on-call duties, and a high volume of emergency cases. Physical strain, such as prolonged standing in lead aprons and intense screen time, adds to the burden (Table 1).

**Table 1:** Comprehensive framework of barriers and facilitators for women in Interventional Radiology

Topic	Aspect	Key focus points
Demands and challenges	Clinical and structural challenges	Emergency workload; long hours; procedural complexity; physical strain (lead aprons); inadequate anesthesia and staff support; demands for multitasking (procedures and post-procedure care, consultations, diagnostic responsibilities, etc.)
	Radiation exposure	Fertility concerns; cumulative exposure; misconceptions about risk during pregnancy; poorly fitting protective gear
Barriers to gender equity	Structural and cultural limitations	Underrepresentation in societies and leadership; implicit bias; caregiving burden; difficulty advancing academic rank; inequitable leave policies
Burnout challenge	Psychological and systemic contributors	Correlation with female gender; excessive hours; emotional labor; conflicts with diagnostic radiology; lack of recognition
Methods to foster balance	Institutional and personal interventions	Flexible work models (part-time, job-sharing); parental leave; ergonomic support; better scheduling; wellness programs; AI-based workflow tools
	Mentorship and networking	Access to female mentors; shared experiences; peer support; knowledge-sharing platforms
	Promotion and leadership	More women in decision-making roles; development of inclusive policies; leadership role models; visibility of women’s success in IR
	Interdisciplinary integration	Enhanced communication; mutual respect; recognition from surgical and medical specialties
	Career advancement opportunities and continuing education	Flexible access to conferences and courses; online learning options; support for maintaining credentials during career breaks
	Leadership as catalyst	Modeling work–life balance; empowering diverse teams; promoting mental health; leadership accountability and evaluation

Beyond procedural demands, IRs also manage consultations and post-procedural care, requiring strong multitasking and time management. These challenges are compounded by a persistent shortage of adequately staffed IR teams and limited access to anesthetic support, as evidenced by a recent evaluation of IR services across England and Wales [3]. Despite rising demand, staffing shortages and limited anesthetic support continue to challenge the field, with nearly 29% of IRs reportedly working over 60 h per week [4, 5].

Radiation exposure in IR is another significant concern [6]. The use of ionizing radiation in interventional radiology procedures can present inherent risks to practitioners and specifically for women during childbearing age, highlighting the need for rigorous adherence to safety protocols [7]. However, studies have shown that the risk of radiation is minimal during fluoroscopic procedures and the risk to the fetus is grossly exaggerated [8]. Nevertheless, and over time, cumulative radiation exposure coupled with the physical strain of wearing lead shielding can lead to significant health concerns [9]. Notably, occupational radiation exposure has been identified as a potential obstacle to achieving gender equity in interventional specialties. Female interventional cardiologists are more likely than their male counterparts to modify their career paths or training choices to minimize radiation exposure [10]. Addressing these obstacles with precise guidelines and a clear consensus by the IR societies could help alleviate any fear women IRs face.

## Barriers to Gender Equity in Interventional Radiology

Women in IR encounter distinct challenges shaped by systemic, cultural, and personal factors, contributing to their underrepresentation and hindering both professional advancement and personal fulfillment. With women making up only 12% of CIRSE members and just two holding positions on its predominantly male committee [11], the lack of representation fosters isolation, limits access to mentorship, and perpetuates biases that question women’s technical skills and dedication to the field. Despite growing awareness, women remain underrepresented in leadership roles across academic, clinical, and organizational domains—a disparity driven by implicit bias, fewer networking opportunities, and the ongoing struggle to balance career and personal responsibilities, especially during childbearing years. To note the upward movement of these disparities, with the gender gap becoming wider in terms of advancing rank (i.e., Associate Professor to Professor) [12].

Balancing the demanding hours of IR with family responsibilities poses a significant challenge. Women often bear a disproportionate share of caregiving duties, making the irregular hours, frequent on-call shifts, and unpredictability of IR more difficult to manage. European women, on average, dedicate about 4.5 h per day to unpaid work, compared to 2.5 h for men, with Greece and Italy leading on the gender gap in unpaid domestic work [13, 14]. While this imbalance affects many women across all professions, the uniquely intense and high-pressure nature of IR amplifies the difficulty of achieving a sustainable work–life balance, making it particularly challenging to navigate for those with substantial family responsibilities.

Furthermore, the physical and systemic demands of IR present unique challenges, particularly for women. Prolonged use of heavy lead aprons, frequently designed for men, can be not only physically taxing, but also responsible for radiation exposure of the axillary breast tissue of female interventional radiologists due to the unproper equipment [15]. Additionally, implicit biases questioning women’s competence, leadership, or commitment—especially during childbearing years—can lead to inequities in career advancement, leadership roles, and research funding. Combined with limited schedule flexibility and a male-dominated perception of the field, these factors may deter women from entering IR or create barriers to reentry after career breaks. Addressing these issues is vital for fostering a more inclusive and supportive IR environment.

## The Burnout Challenge

The notion of the selfless physician, constantly on call and prioritizing patients above all else, has been idealized for decades—depicted in memoirs of legendary surgeons and perpetuated by medical training cultures that equate exhaustion with commitment. However, the reality is far more complex. Physicians frequently face burnout, mental fatigue, and even physical illness, with these challenges varying across specialties. IRs, for instance, experience burnout at significantly higher rates than diagnostic radiologists (54–61%) and surgeons (40%). Studies show that 71.9% of IRs encounter at least one manifestation of burnout during their careers, with contributing factors including female gender (2.4 times higher odds of burnout) and working more than 80 h per week—an all-too-common scenario in under-staffed IR departments [5]. One explanation is that women physicians spend more time per patient and engage more in emotionally focused communication. They also tend to spend more time on electronic health records outside of work hours. These factors increase their workload and emotional labor, heightening the risk of burnout [16]. To note, that excessively long working hours are in violation of European labor laws that apply equally to both male and female staff members.

Burnout among radiologists, including IRs, is a multifactorial issue driven by systemic challenges, interpersonal dynamics, and professional stressors [17]. IRs face unique challenges related to their triple everyday role with procedural, diagnostic, and peri-procedural patient care responsibilities, which can be very demanding, particularly in resource-limited practices. Furthermore, administrative pressures, such as meeting productivity benchmarks (ranging from the number of patients seen, the procedures performed or even to the revenue generated) and managing extensive bureaucratic tasks, act as additional drivers of stress. Contentious relationships with diagnostic radiologists within the same practice further exacerbate professional dissatisfaction and isolation. But perhaps even more troubling for IRs is the lack of recognition and respect from other medical and surgical subspecialties. Strained relationships with ancillary staff and colleagues in multidisciplinary teams create barriers to effective collaboration, compounding emotional exhaustion [18].

## Strategies for Striking the Balance

Improving work conditions and achieving a better work–life balance for women in IR requires addressing the inherent challenges of the specialty through targeted interventions.

One key strategy to achieve better work–life balance is the introduction of more flexible scheduling. This could involve job-sharing arrangements, where two professionals split the responsibilities of one full-time position. Flexible scheduling, especially for women with children or caregiving responsibilities, can allow them to maintain their careers without compromising their personal lives. Hospitals and academic centers that embrace such flexibility can significantly improve job satisfaction and retention among female IRs. Although part-time work may not always be feasible due to organizational constraints or specialty demands, efforts should be made to make it an option wherever possible. With the projected IR workforce shortage over the next decade, offering part-time or flexible arrangements could become a crucial tool for recruitment and retention, ensuring a sustainable and diverse IR workforce.

Furthermore, institutions should try to promote a family-friendly and supportive environment with policies such as paid parental leave, lactation support [19], and predictable on-call schedules to help individuals balance personal and professional demands. While maternity leave and social services vary by country, these policies are essential for supporting work–life balance. Wellness programs that address mental health, stress management, and physical well-being also support a healthier work–life dynamic. Confidential counseling services, mindfulness and meditation workshops, and physician wellness committees are some examples; programs that are already implemented in leading radiology centers, demonstrating their effectiveness in improving physician well-being and fostering a more supportive work environment [20]. Furthermore, ergonomic improvements in the workplace such as lightweight lead aprons that are better adapted to the female morphology, along with access to nutritious meal options, can significantly support physical well-being.

A more efficient organization of workflow is essential to alleviate pressure. Research by Foo et al. demonstrated that separating inpatient and outpatient services enhances role clarity. This division allows physicians assigned to outpatient care to maintain greater control over their schedules, free from the interruptions of emergent cases, and unpredictable add-on procedures [21]. Streamlining scheduling systems to distribute on-call duties more evenly, ensuring predictable work hours, and allocating adequate time for recovery between shifts can significantly reduce the strain of unpredictable and long hours. Implementing team-based models that optimize task delegation and prioritize procedural assignments based on expertise can improve productivity while preventing individual overload. Both the European and American IR societies emphasize the importance of adequate staffing, recommending a minimum of two to three nonphysician personnel—including at least one registered nurse—per procedure room, with additional support required for complex cases and off-hours coverage [22, 23]. Additionally, adopting advanced technologies, such as AI-driven scheduling tools, and automated documentation systems, can help reduce administrative burdens, allowing IRs to focus on patient care. The intelligent application of advanced imaging softwares can greatly improve efficiency, but it is crucial to ensure they complement the expertise of the interventionalist rather than complicate the workflow [24, 25].

Prioritizing key responsibilities and delegating less critical tasks has proven essential in IR. For instance, radiology technicians can handle tasks like setting up equipment or placing PICC lines, allowing interventionalists to focus on the procedure. A strong support system is vital, and this can be built by fostering teamwork and respect among all staff members. Involving everyone—physicians, nurses, and technicians—in the care plan ensures better coordination. Nonmedical staff, such as radiology technicians, can contribute more effectively when included in multidisciplinary care meetings and provided with continuous learning opportunities, like attending scientific congresses or training sessions.

Achieving work–life balance in IR requires reducing stress and replenishing energy—physically, emotionally, and spiritually. Pursuing meaningful goals, like training for a marathon or advocating for gender diversity, enhances resilience. This concept, known as ‘Ikigai’ in Japanese culture, is linked to well-being [26]. While vital for IR practitioners, these strategies benefit all healthcare professionals facing similar demands, fostering fulfillment and longevity.

Women can benefit greatly from mentorship programs that connect them with female senior IRs who have navigated similar challenges [27–30]. These mentors can offer career advice, provide insight, tips, and tricks to help women lead with confidence, share strategies for managing work–life balance, and provide emotional support. Additionally, fostering networking opportunities for women can help create a community of peers who understand the unique challenges women face in this field, helping to mitigate feelings of isolation.

Another essential step forward would be the promotion of women to leadership positions. Having more women in positions of power will allow for the development of policies that reflect the needs of a diverse workforce. It will provide role models for younger women entering the profession, demonstrating that success and balance are achievable.

Given the high-pressure nature of IR, it is crucial for both men and women to prioritize wellness and stress management. Regular exercise, mindfulness, and meditation can help mitigate stress. Fostering a supportive work culture that encourages mental health, time off, and open discussions about stress is essential for balancing career and personal well-being. Regular exercise reduces burnout risk by 44% [31]. Engaging in hobbies or creative outlets can recharge energy, and occasionally declining extra responsibilities is key to avoiding burnout and ensuring long-term well-being.

Interventional radiologists are vital to patient care, yet they may sometimes feel isolated within the broader medical team. To address this, it is essential to foster integration by promoting open communication and collaboration with other specialties. Ensuring that interventional radiologists are recognized and valued in multidisciplinary meetings enhances both professional satisfaction and job fulfillment. Feeling like an integral part of the medical team not only improves morale but also supports better patient outcomes through a more coordinated approach to care.

Finally, we should strain the importance of continuing education and professional development. Attending conferences or pursuing further certifications can help women stay up to date with their skills while managing family life. Virtual learning options or flexible schedules for these career opportunities are essential for women with caregiving responsibilities.

## Leadership as a Catalyst for Work–Life Balance in Interventional Radiology

Strong leadership is crucial in fostering work–life balance and professional fulfillment for women in IR [32–34]. Effective leaders promote flexibility, inclusivity, and understanding, advocating for policies like equitable workloads, flexible schedules, and parental leave to support their teams. They also empower women by mentoring and encouraging them to pursue leadership roles, addressing gender disparities in the field.

Leaders must recognize the unique skills and motivations of each team member, ensuring that at least 20% of their time is dedicated to meaningful activities, such as mentorship, advanced procedures, or community outreach. This balance significantly reduces burnout and enhances career satisfaction [35]. Supervisors who prioritize mental health, foster open communication, and model healthy work–life integration set a powerful example, boosting team morale and retention.

On the contrary, when leaderships fail to meet these standards, organizations should take decisive actions. Just as financial underperformance in the industry warrants leadership changes, consistently low leadership behavior scores, despite adequate coaching, signal the need for new leadership to ensure the team thrives under effective guidance.

## The Path Forward

While achieving work–life balance in IR is challenging, institutions, mentors, and individuals can make a significant difference in improving the experience for women in the field. As more women enter the specialty, it is crucial to advocate for policies and practices that support them, fostering a diverse, equitable, and sustainable workforce. By promoting flexible work options, mentorship, and addressing gender bias, the field can become more inclusive and supportive, benefiting both women and the specialty as a whole.

In conclusion, the journey of women in interventional medicine has come a long way, from navigating the demanding interventional theater to finding harmony in their personal lives. Striking this balance remains challenging, but with support, flexibility, and determination it can be achieved. As barriers continue to be broken down and more inclusive environments are created, the path for women in IR can be both rewarding and fulfilling. By fostering respect and understanding, we can ensure that women have the opportunity to thrive in both their careers and their personal lives, ultimately shaping a more diverse and dynamic future for the field.
